# Supplementary material for: Food Literacy and Dietary Intake in German Office Workers: A Longitudinal Intervention Study
Source: Int J Environ Res Public Health. 2022 Dec 9;19(24):16534. doi: 10.3390/ijerph192416534 (PMC9779084; doi:10.3390/ijerph192416534)
Supplement: Supplementary file 1 [file ijerph-19-16534-s001.zip › Document S1 Declaration of informed consent.pdf]

Declaration of informed consent - Evaluation of the occupational health management programs at Siemens AG – Evaluation Project

Background on the evaluation project

Siemens attaches great importance to protecting and promoting the health, motivation and performance of its employees. In this context, you, as a part of Siemens, have decided to participate in a three-week workplace health promotion program.

In order to achieve a long-lasting effect of our prevention offers, to continuously improve them and to ensure their effectiveness, we ask you to participate in the accompanying study "Evaluation of the occupational health management programs at Siemens AG – Evaluation Project", which is being conducted independently by the Faculty of Sports and Health Sciences at the Technical University of Munich. Participation in the Evaluation Project is voluntary and can be terminated any time without specific cause and without consequences from the termination. Details on the planned surveys and on data protection can be found on the following pages.

Data collection procedure

The study includes four measurement time points and we would like to ask you to participate at all four time points. At the beginning (T0), at the end (T1), six months (T2) and 18 months (T3) after the end of the prevention offer, we will invite you to participate in a survey in a paper and/or online version. In addition, we will use a pseudonym to combine your information from the survey across the different time points. The scientist of the Faculty of Sports and Health Sciences at the Technical University of Munich will conduct the survey. No other party will be involved in the development of the survey or have any access to the data collected from you. The following data will be collected over the measurement period:

Demographic data: Age, gender, marital status, education level

Health behaviour: Physical activity, dietary behaviour, sleep behaviour

Health literacy: physical activity-related health literacy, food literacy

Health status: health-related quality of life, symptoms of chronic stress

If you would like to receive further information before the start of the study, please contact the project leader Dr. Ulrich Birner (ulrich.birner@siemens.com), HR EHS DE EC E at Siemens AG, Siemens Health Management (HR EHS DE HM), Otto Hahn Ring 6, 81739 München („Siemens Health Management") or the responsible researchers, Simon Blaschke (simon.blaschke@tum.de), at the Chair of Sports and Health Didactics at the Faculty of Sports and Health Sciences at TU Munich.

Data analysis and Reporting

The processing and analysis of the survey data are carried out on the basis of pseudonymous data, i.e. all data directly identifying your person are replaced by an identification code (pseudonymised) during the study. For this purpose, at the beginning of the survey, you will have the opportunity to create a code on page 4 consisting of a multi-digit combination of letters and numbers that will replace your name and that will be assigned to your data. The processing of your data for the

evaluation project over different points in time is then carried out exclusively using this letter and number combination. The results and the reporting of the evaluation are anonymous, i.e. no conclusions can be drawn about individual persons from the evaluation reporting or results.

Purpose of the processing

The data described before will be used on the basis of this consent exclusively for the aim of the evaluation project to examine the implementation and efficacy of the prevention offers at Siemens AG by the Chair of Sports and Health Didactics at the Faculty of Sports and Health Sciences at TU Munich within the scientific aim of the evaluation project. Processing for other purposes is excluded. After completion of the data collection, the collected data will be used exclusively in pseudonymised form for the analysis of the research project and for archiving the research results. In the event of publication in a scientific paper, the data will not reveal who participated in this research. All personal data is subject to the Data Protection Act.

Recipient of the data collected

The evaluation project is conducted by the Technical University of Munich, Faculty of Sport and Health Sciences, Georg-Brauchle-Ring 60/62, 80992 Munich ("TU Munich"). For this purpose, your data will be collected and transmitted to the TU Munich in pseudonymised form, i.e. under the letter or number combination you have assigned, but without your name or any other confidential detail.

Retention periods

Your data will be safely stored and supervised by Simon Blaschke at the Faculty of Sport and Health Science at the Chair of Sport and Health Didactics. Data will be deleted after expiry of the statutory deletion periods in accordance with the specifications for "safeguarding good scientific practice" after a retention period of 10 years.

Consent

I hereby consent to TU Munich for collecting and processing my personal data to the extent and for the purposes described above. I am aware that the consent includes so-called special types of personal data, i.e. data that allow conclusions to be drawn about my state of health and are subject to medical confidentiality pursuant to §203 of the German Criminal Code.

I am aware that the declaration of my consent is voluntary and that I have the right to revoke it at any time with effect for TU Munich. To do so, simply send a message to the supervising researcher, Simon Blaschke, (simon.blaschke@tum.de). You also have the right to receive information about the data concerning you, also in the form of a free copy. Furthermore, you can request the correction or deletion of your data.

First name, last name: ____

Signature: ____

Place, Date: ____

Further important information – Right to information, correction, deletion or restriction of your personal data, right to object and right to data portability

Under applicable data protection law, you may have the right to:

- Request confirmation as to whether TU Munich are processing personal data about you and to receive information about the personal data processed TU Munich and other information
- Request the rectification of inaccurate personal data
- Request the erasure of personal data processed
- Request the restriction of the processing of personal data
- Obtain personal data that you have provided in a structured, commonly used and machine-readable format or request that the personal data be transferred to a third party
- Object to the processing of your personal data.

You can assert the aforementioned data subject rights with effect against TU Munich at simon.blaschke@tum.de or at datenschutz@siemens.com

Additional Contact

The Siemens Data Protection Organization can also provide support in all matters relating to data protection. Complaints can also be made to the Siemens data protection organization and the rights mentioned in this consent can be asserted. The Siemens Data Protection Organization can be contacted at datenschutz@siemens.com.

The Siemens data protection organisation will always endeavour to address and remedy your enquiry and complaint. In addition to contacting the Siemens data protection organisation, you can also contact the competent data protection supervisory authority with your enquiry or complaint at any time.

The competent data protection authority is:

Bayerisches Landesamt für Datenschutzaufsicht (BayLDA)

Promenade 27

91522Ansbach, Germany

Coding instructions – Person ID or pseudonymisation

We now ask you to create a code from a multi-digit combination of letters and numbers that will replace your name and be assigned to your data. You will be given instructions on how your code is made up at each subsequent interview, so that you do not have to remember it

a) The first two letters of your mother's maiden name (or a person who is closest to a mother for you): e.g. Erika Mustermann née Schmidt
   First two letters: _______

b) The last two letters of your father's (first) name (or a person closest to a father for you): e.g. MichaEL
   First two letters: _______

c) The day of your date of birth (prefix single-digit numbers with "0"): e.g. 9.1.1976 = 09
   Day of birth: _______

d) The number of letters of your (first) name (put a "0" in front for single-digit numbers): e.g. Markus = 06
   Number of letters: _______

The code in this example would be: SCEL0906
