# Supplementary material for: Food Literacy and Dietary Intake in German Office Workers: A Longitudinal Intervention Study
Source: Int J Environ Res Public Health. 2022 Dec 9;19(24):16534. doi: 10.3390/ijerph192416534 (PMC9779084; doi:10.3390/ijerph192416534)
Supplement: Supplementary file 1 [file ijerph-19-16534-s001.zip › Document S2 Overview of item responses.pdf]

Overview of Item Responses

Food Literacy	T0	T1	T2	T3
**SFLQ_01**	3.2 (0.7)	3.5 (0.5)	3.6 (0.5)	3.5 (0.6)
**SFLQ_02_average**	3.7 (0.8)	3.4 (0.5)	3.9 (0.8)	3.9 (0.8)
**SFLQ_02a**	3.8 (1.4)	3.4 (0.9)	4.2 (1.0)	4.2 (0.9)
**SFLQ_02b**	3.3 (1.1)	3.8 (0.9)	3.6 (1.0)	3.7 (1.1)
**SFLQ_02c**	3.7 (1.5)	3.9 (1.1)	3.8 (1.3)	3.8 (1.3)
**SFLQ_02d**	4.1 (1.2)	2.9 (1.1)	4.1 (1.2)	4.0 (1.3)
**SFLQ_02e**	3.8 (1.1)	3.0 (1.1)	4.0 (1.1)	3.9 (1.1)
**SFLQ_03**	2.7 (1.5)	3.7 (1.2)	3.8 (1.0)	3.8 (1.1)
**SFLQ_04**	2.4 (0.9)	3.0 (0.7)	3.1 (0.7)	3.1 (0.7)
**SFLQ_05**	2.2 (0.8)	2.8 (0.8)	2.9 (0.8)	2.9 (0.7)
**SFLQ_06_average**	2.3 (0.8)	2.8 (0.5)	2.8 (0.7)	2.6 (0.7)
**SFLQ_06a**	2.4 (0.9)	3.0 (0.8)	3.0 (0.8)	2.8 (0.8)
**SFLQ_06b**	2.3 (1.0)	2.6 (0.8)	2.6 (0.8)	2.5 (0.9)
**SFLQ_07**	2.3 (1.3)	2.8 (0.8)	2.9 (1.2)	2.8 (1.2)
**SFLQ_08**	3.2 (1.1)	4.1 (0.8)	4.0 (0.9)	3.9 (0.9)
**SFLQ_09**	2.4 (0.6)	2.8 (0.7)	2.9 (0.6)	2.9 (0.7)
**SFLQ_10**	2.6 (0.7)	3.0 (0.7)	3.1 (0.7)	3.0 (0.6)
**SFLQ_11**	2.9 (0.6)	3.1 (0.6)	3.2 (0.5)	3.1 (0.5)
**SFLQ_12**	2.9 (0.7)	3.3 (0.6)	3.3 (0.5)	3.2 (0.5)

Dietary Intake	T0	T1	T2	T3
**FFQ_01_Meat**	1.1 (0.6)	1.4 (0.6)	1.3 (0.5)	1.4 (0.6)
**FFQ_02_Sausages**	1.1 (0.5)	1.1 (0.8)	1.2 (0.6)	1.3 (0.6)
**FFQ_04_Fish**	1.3 (0.8)	1.8 (0.4)	1.5 (0.7)	1.6 (0.7)
**FFQ_05_Potatoes**	0.5 (0.5)	0.3 (0.4)	0.4 (0.5)	0.4 (0.5)
**FFQ_06_Pasta**	0.4 (0.5)	0.2 (0.4)	0.3 (0.5)	0.4 (0.5)
**FFQ_07_Rice**	0.1 (0.3)	0.2 (0.4)	0.2 (0.4)	0.2 (0.4)
**FFQ_08_Salad**	1.1 (0.7)	1.8 (0.5)	1.2 (0.7)	1.2 (0.7)
**FFQ_09_Vegetables**	0.8 (0.7)	1.4 (0.7)	1.0 (0.7)	1.0 (0.7)
**FFQ_10_Fruit**	1.1 (0.8)	1.5 (0.7)	1.3 (0.8)	1.3 (0.8)
**FFQ_11_Chocolate**	0.7 (0.7)	1.8 (0.5)	0.8 (0.7)	0.7 (0.7)
**FFQ_12_Cake**	0.8 (0.7)	1.8 (0.5)	1.0 (0.6)	0.8 (0.7)
**FFQ_14_Salty snacks**	1.1 (0.7)	1.9 (0.4)	1.3 (0.6)	1.2 (0.7)
**FFQ_16_Wholegrain bread**	0.9 (0.7)	1.2 (0.8)	0.9 (0.7)	0.9 (0.7)
**FFQ_17_Oats, Cereals**	1.1 (0.8)	1.4 (0.8)	1.4 (0.8)	1.3 (0.9)
**FFQ_20_Eggs**	1.6 (0.5)	1.6 (0.6)	1.6 (0.6)	1.6 (0.6)

Note: Values are presented as Mean (SD).
