# Supplementary material for: Food Literacy and Dietary Intake in German Office Workers: A Longitudinal Intervention Study
Source: Int J Environ Res Public Health. 2022 Dec 9;19(24):16534. doi: 10.3390/ijerph192416534 (PMC9779084; doi:10.3390/ijerph192416534)
Supplement: Supplementary file 1 [file ijerph-19-16534-s001.zip › Document S3 Survey for the evaluation of the WHPP at Siemens.pdf]

Professur für Sport- und Gesundheitsdidaktik
Fakultät für Sport- und Gesundheitswissenschaften
Technische Universität München
Uptown München-Campus D
Georg-Brauchle-Ring 60/62
80992 München

Befragung zur Evaluation der Siemens-Präventionsangebote

Survey for the evaluation of WHPPs at Siemens

Liebe Teilnehmerin / lieber Teilnehmer an dem Gesundheitstraining oder Gesundheitsseminar,
Dear participant in the health training or health seminar,

der Schutz und die Förderung von Gesundheit, Motivation und Leistungsfähigkeit der Mitarbeitenden haben bei Siemens einen hohen Stellenwert. Mit Ihrer Einwilligung, an dem Projekt **„Evaluation der Siemens-Präventionsangebote“** teilzunehmen, leisten Sie einen wesentlichen Beitrag zur Sicherstellung der bestmöglichen Qualität dieser Maßnahmen zur Gesundheitsförderung bei Siemens.

Nutzen

Protecting and promoting the health, motivation and performance of employees is a top priority at Siemens. By agreeing to take part in the survey "Evaluation of WHPPs at Siemens ", you are making a significant contribution to ensuring the best possible quality of these health promotion measures at Siemens.

Rationale

Das Projekt wird durch die Fakultät für Sport- und Gesundheitswissenschaften der Technischen Universität München (TUM) durchgeführt. Es sieht Befragungen vor und nach den einzelnen Maßnahmen in den Präventionsangeboten vor. Dabei werden alle Gesundheitsthemen angesprochen, die in diesen Präventionsangeboten eine zentrale Rolle spielen.

Organisation

The project is conducted by the Faculty of Sport and Health Sciences at the Technical University of Munich (TUM). It provides for surveys before and after the individual measures in the prevention programmes. All health topics that play a central role in these prevention programmes are addressed.

Organization

Der folgende Fragebogen enthält eine Reihe von Aussagen zu Ihrer Gesundheit. Ihre Antworten geben uns zum einen genaueren Einblick in die Wirksamkeit der verschiedenen Präventionsangebote. Zum anderen erfahren wir so mehr über Ihre Bedürfnisse und Wünsche, um diese bei der zukünftigen Gestaltung der Angebote zu berücksichtigen.

Ziele

The following questionnaire contains a number of statements about your health. On the one hand, your answers give us a more precise insight into the effectiveness of

Aims

the various prevention services. On the other hand, we learn more about your needs and wishes in order to take them into account in the future design of the WHPPs.

Hinweise zum Ausfüllen

Zu jeder Aussage finden Sie mehrere Kästchen mit Antwortmöglichkeiten. Kreuzen Sie bitte das Kästchen an, das dem Ausmaß Ihrer Zustimmung am besten entspricht. Scheuen Sie sich nicht, auch extreme Werte anzukreuzen, wenn dies für Sie zutrifft. Es gibt dabei keine ‚richtigen' oder ‚falschen' Angaben – es kommt auf Ihr Erleben an. **Die Beantwortung ist freiwillig. Das Ausfüllen dauert ca. 10 Minuten.**

Advice on survey

For each statement you will find several boxes with possible answers. Please tick the box that best corresponds to your level of agreement. Do not be afraid to tick extreme values if this applies to you. There are no 'right' or 'wrong' answers - it all depends on your experience. Answering the questionnaire is voluntary. It takes about 10 minutes to complete.

Anonymität

Die Verarbeitung Ihrer Daten für das Evaluationsprojekt über verschiedene Zeitpunkte erfolgt ausschließlich unter Verwendung Ihres pseudonymisierten Kennworts. Die Ergebnisse der Auswertung sind anonym, d.h. aus den Auswertungsergebnissen können keinerlei Rückschlüsse auf einzelne Personen mehr gezogen werden.

Data privacy

The processing of your data for the evaluation project over various points in time will be carried out exclusively using your pseudonymised code word. The results of the evaluation are anonymous, i.e. no conclusions can be drawn about individual persons from the evaluation results.

Soziodemografische Fragen

Sociodemographic Questions

1. Welches Geschlecht haben Sie?
What gender are you?

Weiblich *Female*	Männlich *Male*	Divers *Diverse*
☐	☐	☐

2. Bitte geben Sie Ihr Alter in Jahren an: ___________ Jahre
Please state your age in years: ___________ *years*

3. Welchen Familienstand haben Sie?
What is your marital status?

- ☐ Ledig
Single
- ☐ Verheiratet
Married
- ☐ Geschieden
Divorced
- ☐ Verwitwet
Widowed
- ☐ Sonstiges (Bitte geben Sie dies an): ____________________
Other (Please specify) ____________________

4. Was ist Ihr höchster Bildungsabschluss?
What is your highest level of education?

- ☐ kein Schulabschluss
No school-leaving qualification
- ☐ Haupt/Mittelschulabschluss
Lower secondary school
- ☐ Mittlere Reife/Realschulabschluss
General secondary school certificate
- ☐ Abitur/Gymnasialer Schulabschluss
General qualification for university entrance
- ☐ Abgeschlossene Ausbildung
Substantive vocational training
- ☐ Fachhochschulabschluss
Polytechnic university degree
- ☐ Hochschule (Bachelor)
Bachelor’s degree
- ☐ Hochschule (Master, Magister, Diplom)
Master’s degree
- ☐ Hochschule Promotion
Doctoral degree
- ☐ Sonstiges (Bitte geben Sie dies an): ____________________
Others (Please specify): ____________________

Nächste Seite ...

Ernährung

Dietary intake

Bitte geben Sie für jedes der folgenden Lebensmittel bzw. Getränke an, wie häufig Sie dieses **in den letzten 4 Wochen** verzehrt haben. Denken Sie bei der Beantwortung an Ihr gewöhnliches Ernährungsverhalten.
For each of the following foods or beverages, please indicate how often you have consumed it in the last 4 weeks. When answering, please keep in mind your usual dietary habits.

	Lebensmittelgruppe<br>*Food groups*	Nie<br>*Never*	1x im Monat oder seltener<br>*Once a month or less fre-quently*	Mehr-mals im Monat<br>*Several times a month*	Etwa 1x in der Woche<br>*About once a month*	Mehr-mals in der Woche<br>*More than once a week*	Fast täglich<br>*Almost daily*
5.	Fleisch (ohne Wurstwaren)<br>*Meat (without sausages)*	0	1	2	3	4	5
6.	Wurstwaren, Schinken<br>*Sausages, Ham*	0	1	2	3	4	5
7.	Geflügel<br>*Poultry*	0	1	2	3	4	5
8.	Fisch<br>*Fish*	0	1	2	3	4	5
9.	Kartoffeln<br>*Potatoes*	0	1	2	3	4	5
10.	Teigwaren<br>*Baking goods*	0	1	2	3	4	5
11.	Reis<br>*Rice*	0	1	2	3	4	5
12.	Salat<br>*Salad*	0	1	2	3	4	5
13.	Gemüse<br>*Vegetables*	0	1	2	3	4	5
14.	Obst<br>*Fruit*	0	1	2	3	4	5
15.	Schokolade<br>*Choclate*	0	1	2	3	4	5
16.	Kuchen, Gebäck, Kekse<br>*Cake and Pastry*	0	1	2	3	4	5
17.	Süßwaren<br>*Candy*	0	1	2	3	4	5
18.	Salzige Knabbereien<br>*Salty snacks*	0	1	2	3	4	5

	Lebensmittelgruppe<br>*Food groups*	Nie<br>*Never*	1x im Monat oder seltener<br>*Once a month or less frequently*	Mehrmals im Monat<br>*Several times a month*	Etwa 1x in der Woche<br>*About once a month*	Mehrmals in der Woche<br>*More than once a week*	Fast täglich<br>*Almost daily*
19.	Weißbrot<br>*White bread*	0	1	2	3	4	5
20.	Vollkornbrot<br>*Wholegrain bread*	0	1	2	3	4	5
21.	Haferflocken, Müsli, Cornflakes<br>*Oatmeal and cereals*	0	1	2	3	4	5
22.	Quark, Joghurt, Dickmilch<br>*Curd cheese, yoghurt, soured milk*	0	1	2	3	4	5
23.	Käse<br>*Cheese*	0	1	2	3	4	5
24.	Eier<br>*Eggs*	0	1	2	3	4	5
25.	Milch<br>*Milk*	0	1	2	3	4	5
26.	Obstsäfte<br>*Juices*	0	1	2	3	4	5
27.	Cola, Limonade, Eistee<br>*Softdrinks and ice tea*	0	1	2	3	4	5
28.	Mineralwasser<br>*Sparkling water*	0	1	2	3	4	5
29.	Diätlimonade, sonst. Diätgetränke<br>*Dietary drinks*	0	1	2	3	4	5

Nächste Seite ...[redacted]

Lebensmittelkompetenz
Food Literacy

30. Wenn ich Fragen zu gesunder Ernährung habe, weiß ich wo ich mich informieren kann.
When I have questions on healthy nutrition, I know where I can find information on this issue.

trifft voll und ganz zu *Agree strongly*	trifft eher zu *Agree*	trifft eher nicht zu *Disagree*	trifft überhaupt nicht zu *Disagree strongly*	ich habe keine Erfahrung damit *I have no experience with this*
☐	☐	☐	☐	☐

Wie gut verstehen Sie im Allgemeinen folgende Informationen? Gemeint ist dabei, die Verständlichkeit und nicht die inhaltliche Qualität der Information. *Bitte kreuzen Sie pro Zeile je eine Antwort an*
In general, how well do you understand the following types of nutritional information?

		Sehr gut *Very good*	Gut *Good*	Mittelmäßig *Mediocre*	Schlecht *Bad*	Sehr schlecht *Very bad*	Nutze ich nicht *I do not make use of this kind of information*
31.	Informationsbroschüren über Ernährung *Nutrition information leaflets*	☐	☐	☐	☐	☐	☐
32.	Informationen auf Lebensmittelverpackungen *Food label information*	☐	☐	☐	☐	☐	☐
33.	Fernseh- oder Radiosendungen über Ernährung *TV or radio program on nutrition*	☐	☐	☐	☐	☐	☐
34.	Mündliche Empfehlungen von Fachpersonen zur Ernährung *Oral recommendations regarding nutrition from professionals.*	☐	☐	☐	☐	☐	☐
35.	Ratschläge zur Ernährung von Familienmitgliedern, Freunden oder Bekannten *Nutrition advice from family members or friends*	☐	☐	☐	☐	☐	☐

36. Wie gut kennen Sie die Lebensmittelpyramide der deutschen Gesellschaft für Ernährung?
How familiar are you with the German Food Pyramid?

sehr schlecht *Very bad*	Schlecht *Bad*	Mittelmäßig *Mediocre*	Gut *Good*	sehr gut *Very good*
☐	☐	☐	☐	☐

37. Ich kenne die offiziellen Empfehlungen zum Früchte- und Gemüseverzehr der dt. Gesellschaft für Ernährung.

I know the official German recommendations about fruit and vegetable consumption.

trifft voll und ganz zu *Strongly agree*	trifft eher zu *Agree*	trifft eher nicht zu *Disagree*	trifft überhaupt nicht zu *Strongly agree*
☐	☐	☐	☐

38. Ich kenne die offiziellen Empfehlungen zur Salzaufnahme der deutschen Gesellschaft für Ernährung.

I know the official Swiss recommendations about salt intake

trifft voll und ganz zu *Strongly agree*	trifft eher zu *Agree*	trifft eher nicht zu *Disagree*	trifft überhaupt nicht zu *Strongly disagree*
☐	☐	☐	☐

39. An einem normalen Tag: Wie leicht oder schwer fällt es Ihnen zu Hause eine ausgewogene Mahlzeit zusammenzustellen

Think about a usual day: how easy or difficult is it for you to compose a balanced meal at home?

Sehr leicht *Very easy*	Ziemlich leicht *Easy*	Ziemlich schwer *Hard*	Sehr schwer *Very hard*	Mache ich nicht *Not applicable*
☐	☐	☐	☐	☐

40. Und wie ist es mit dem Essen außer Haus: Wie leicht oder schwer fällt es Ihnen dort, an einem normalen Tag eine ausgewogene Mahlzeit zusammenzustellen?

Think about a usual day: how easy or difficult is it for you to compose a balanced meal at away from home?

Sehr leicht *Very easy*	Ziemlich leicht *Easy*	Ziemlich schwer *Hard*	Sehr schwer *Very hard*	Mache ich nicht *Not applicable*
☐	☐	☐	☐	☐

41. Wie oft konnten Sie in der Vergangenheit jemanden aus Ihrer Familie oder dem Bekanntenkreis bei Fragen zur Ernährung weiterhelfen?

In the past, how often were you able to help your family members or a friend if they had questions concerning nutritional issues?

Nie *Never*	Selten *Seldom*	Gelegentlich *Sometimes*	Oft *Often*	Immer *Always*	Es gab nie Fragen dazu *There have never been any questions*
☐	☐	☐	☐	☐	☐

42. Es gibt heute viele Informationen zum Thema gesunde Ernährung. Wie gut gelingt es Ihnen die für Sie relevanten Informationen auszuwählen?

There is a lot of information available on healthy nutrition today. How well do you manage to choose the information relevant to you?

Sehr schlecht *Very bad*	Schlecht *Bad*	Mittelmäßig *Mediocre*	Gut *Good*	Sehr gut *Very good*	kein Interesse an solchen Informationen *I have not been interested in these issues*

☐	☐	☐	☐	☐	☐

43. Wie leicht fällt es Ihnen zu beurteilen, ob Informationen aus den Medien zum Thema Ernährung vertrauenswürdig sind?

How easy is it for you to judge if media information on nutritional issues can be trusted?

Sehr leicht<br>*Very easy*	Ziemlich leicht<br>*Easy*	Ziemlich schwer<br>*Hard*	Sehr schwer<br>*Very hard*
☐	☐	☐	☐

44. In der Werbung werden Lebensmittel häufig mit Gesundheit in Verbindung gebracht. Wie leicht fällt es Ihnen zu beurteilen, inwiefern die dargestellten Zusammenhänge zutreffen oder nicht?

Commercials often relate foods with health. How easy is it for you to judge if the presented associations are appropriate or not?

Sehr leicht<br>*Very easy*	Ziemlich leicht<br>*Easy*	Ziemlich schwer<br>*Hard*	Sehr schwer<br>*Very hard*
☐	☐	☐	☐

45. Wie leicht fällt es Ihnen einzuschätzen, ob ein Lebensmittel für eine gesunde Ernährung von Bedeutung ist?

How easy is it for you to evaluate if a specific food is relevant for a healthy diet?

Sehr leicht<br>*Very easy*	Ziemlich leicht<br>*Easy*	Ziemlich schwer<br>*Hard*	Sehr schwer<br>*Very hard*
☐	☐	☐	☐

46. Wie leicht fällt es Ihnen einzuschätzen, welchen Einfluss Ihre Essgewohnheiten langfristig auf Ihre Gesundheit haben können?

How easy is it for you to evaluate the longer-term impact of your dietary habits on your health?

Sehr leicht<br>*Very easy*	Ziemlich leicht<br>*Easy*	Ziemlich schwer<br>*Hard*	Sehr schwer<br>*Very hard*
☐	☐	☐	☐

Herzlichen Dank für Ihre Teilnahme!
