# Supplementary material for: Food Literacy and Dietary Intake in German Office Workers: A Longitudinal Intervention Study
Source: Int J Environ Res Public Health. 2022 Dec 9;19(24):16534. doi: 10.3390/ijerph192416534 (PMC9779084; doi:10.3390/ijerph192416534)
Supplement: Supplementary file 1 [file ijerph-19-16534-s001.zip › Document S4 WHPP Schedule.pdf]

<table>
<tr><th>1st Day
(Wednesday)</th><th>2nd Day
(Thursday)</th><th>3rd Day
(Friday)</th><th>4th Day
(Saturday)</th><th>5th Day
(Sunday)</th><th>6th Day
(Monday)</th><th>7th Day
(Tuesday)</th></tr>
<tr><td rowspan="3">7.30 am – 12.00 pm
Arrival of the participants</td><td>7.30 am – 8.00 am
Morning Activation</td><td>7.30 am – 8.00 am
Morning Activation</td><td>7.30 am – 8.00 am
Morning Activation</td><td>7.30 am – 8.00 am
Rest day</td><td>7.30 am – 8.00 am
Morning Activation</td><td>7.30 am – 8.00 am
Morning Activation</td></tr>
<tr><td>8.30 am – 9.30 am
Breakfast</td><td>8.30 am – 9.30 am
Breakfast</td><td>8.30 am – 9.30 am
Breakfast</td><td>8.30 am – 9.30 am
Breakfast</td><td>8.30 am – 9.30 am
Breakfast</td><td>8.30 am – 9.30 am
Breakfast</td></tr>
<tr><td>9.30 am – 11.30 am
Exercise Program</td><td>9.30 am – 11.30 am
Exercise Program</td><td>9.30 am – 11.30 am
Exercise Program</td><td>9.30 am – 11.30 am
Rest day</td><td>9.30 am – 11.30 am
Exercise Program</td><td>9.30 am – 11.30 am
Exercise Program</td></tr>
<tr><td>12.00 pm – 1.00 pm
Lunch</td><td>12.00 pm – 1.00 pm
Lunch</td><td>12.00 pm – 1.00 pm
Lunch</td><td>12.00 pm – 1.00 pm
Lunch</td><td>12.00 pm – 1.00 pm
Lunch</td><td>12.00 pm – 1.00 pm
Lunch</td><td>12.00 pm – 1.00 pm
Lunch</td></tr>
<tr><td>1.00 pm – 2.00 pm
Official Welcoming</td><td>1.00 pm – 2.00 pm
Leisure Time</td><td>1.00 pm – 2.00 pm
Leisure Time</td><td>1.00 pm – 2.00 pm
Leisure Time</td><td rowspan="5">1.00 pm – 6.00 pm
Rest day</td><td>1.00 pm – 2.00 pm
Leisure Time</td><td>1.00 pm – 2.00 pm
Leisure Time</td></tr>
<tr><td rowspan="4">2.00 pm – 6.00 pm
Medical health check</td><td>2.00 pm – 3.00 pm
Presentation “Health risks”</td><td>2.00 pm – 3.00 pm
Presentation “Training Control”</td><td rowspan="4">2.00 pm – 6.00 pm
Hiking Trip</td><td rowspan="4">2.00 pm – 6.00 pm
Workshop „Dietary Intake and Health“</td><td rowspan="2">2.00 pm – 4.00 pm
Exercise Program</td></tr>
<tr><td>3.00 pm – 4.00 pm
Exercise Program</td><td rowspan="2">3.00 pm – 5.00 pm
Exercise Program</td></tr>
<tr><td>4.00 pm – 5.00 pm
Presentation “Health behavior”</td><td>4.00 pm – 5.00 pm
Presentation “Postural Complaints”</td></tr>
<tr><td>5.00 pm – 6.00 pm
Leisure Time</td><td>5.00 pm – 6.00 pm
Leisure Time</td><td>5.00 pm – 6.00 pm
Leisure Time</td></tr>
<tr><td>6.00 pm – 7.00 pm
Dinner</td><td>6.00 pm – 7.00 pm
Dinner</td><td>6.00 pm – 7.00 pm
Dinner</td><td>6.00 pm – 7.00 pm
Dinner</td><td>6.00 pm – 7.00 pm
Dinner</td><td>6.00 pm – 7.00 pm
Dinner</td><td>6.00 pm – 7.00 pm
Dinner</td></tr>
<tr><td>From 7.00 pm
Leisure Time</td><td>From 7.00 pm
Leisure Time</td><td>From 7.00 pm
Leisure Time</td><td>From 7.00 pm
Leisure Time</td><td>From 7.00 pm
Leisure Time</td><td>From 7.00 pm
Leisure Time</td><td>From 7.00 pm
Leisure Time</td></tr>
</table>

<table>
<tr><th>8th Day
(Wednesday)</th><th>9th Day
(Thursday)</th><th>10th Day
(Friday)</th><th>11th Day
(Saturday)</th><th>12th Day
(Sunday)</th><th>13th Day
(Monday)</th><th>14th Day
(Tuesday)</th></tr>
<tr><td>7.30 am – 8.00 am
Morning Activation</td><td>7.30 am – 8.00 am
Morning Activation</td><td>7.30 am – 8.00 am
Morning Activation</td><td>7.30 am – 8.00 am
Morning Activation</td><td>7.30 am – 8.00 am
Rest day</td><td>7.30 am – 8.00 am
Morning Activation</td><td>7.30 am – 8.00 am
Morning Activation</td></tr>
<tr><td>8.30 am – 9.30 am
Breakfast</td><td>8.30 am – 9.30 am
Breakfast</td><td>8.30 am – 9.30 am
Breakfast</td><td>8.30 am – 9.30 am
Breakfast</td><td>8.30 am – 9.30 am
Breakfast</td><td>8.30 am – 9.30 am
Breakfast</td><td>8.30 am – 9.30 am
Breakfast</td></tr>
<tr><td>9.30 am – 11.30 am
Exercise Program</td><td>9.30 am – 11.30 am
Exercise Program</td><td>9.30 am – 11.30 am
Exercise Program</td><td>9.30 am – 11.30 am
Exercise Program</td><td>9.30 am – 11.30 am
Rest day</td><td>9.30 am – 11.30 am
Exercise Program</td><td>9.30 am – 11.30 am
Exercise Program</td></tr>
<tr><td>12.00 pm – 1.00 pm
Lunch</td><td>12.00 pm – 1.00 pm
Lunch</td><td>12.00 pm – 1.00 pm
Lunch</td><td>12.00 pm – 1.00 pm
Lunch</td><td>12.00 pm – 1.00 pm
Lunch</td><td>12.00 pm – 1.00 pm
Lunch</td><td>12.00 pm – 1.00 pm
Lunch</td></tr>
<tr><td>1.00 pm – 2.00 pm
Leisure Time</td><td>1.00 pm – 2.00 pm
Leisure Time</td><td rowspan="2">1.00 pm – 3.00 pm
Leisure Time</td><td>1.00 pm – 2.00 pm
Leisure Time</td><td rowspan="5">1.00 pm – 6.00 pm
Rest day</td><td>1.00 pm – 2.00 pm
Leisure Time</td><td>1.00 pm – 2.00 pm
Leisure Time</td></tr>
<tr><td>2.00 pm – 3.00 pm
Presentation “Health behavior change”</td><td rowspan="2">2.00 pm – 4.00 pm
Exercise Program</td><td rowspan="4">2.00 pm – 6.00 pm
Hiking Trip</td><td rowspan="2">2.00 pm – 4.00 pm
Exercise Program</td><td rowspan="2">2.00 pm – 4.00 pm
Exercise Program</td></tr>
<tr><td rowspan="3">3.00 pm – 6.00 pm
Leisure Time</td><td rowspan="2">3.00 pm – 5.00 pm
Exercise Program</td></tr>
<tr><td>4.00 pm – 5.00 pm
Presentation “Health-enhancing PA”</td><td rowspan="2">4.00 pm – 6.00 pm
Leisure Time</td><td rowspan="2">4.00 pm – 6.00 pm
Leisure Time</td></tr>
<tr><td>5.00 pm – 6.00 pm
Leisure Time</td><td>5.00 pm – 6.00 pm
Leisure Time</td></tr>
<tr><td>6.00 pm – 7.00 pm
Dinner</td><td>6.00 pm – 7.00 pm
Dinner</td><td>6.00 pm – 7.00 pm
Dinner</td><td>6.00 pm – 7.00 pm
Dinner</td><td>6.00 pm – 7.00 pm
Dinner</td><td>6.00 pm – 7.00 pm
Dinner</td><td>6.00 pm – 7.00 pm
Dinner</td></tr>
<tr><td>From 7.00 pm
Leisure Time</td><td>From 7.00 pm
Leisure Time</td><td>From 7.00 pm
Leisure Time</td><td>From 7.00 pm
Individual Coaching</td><td>From 7.00 pm
Leisure Time</td><td>From 7.00 pm
Individual Coaching</td><td>From 7.00 pm
Individual Coaching</td></tr>
</table>

15th Day (Wednesday)	16th Day (Thursday)	17th Day (Friday)	18th Day (Saturday)	19th Day (Sunday)	20th Day (Monday)	21st Day (Tuesday)
**7.30 am – 8.00 am** Morning Activation	**7.30 am – 8.00 am** Morning Activation	**7.30 am – 8.00 am** Morning Activation	**7.30 am – 8.00 am** Morning Activation	**7.30 am – 8.00 am** Rest day	**7.30 am – 8.00 am** Morning Activation	**7.30 am – 8.00 am** Morning Activation
**8.30 am – 9.30 am** Breakfast	**8.30 am – 9.30 am** Breakfast	**8.30 am – 9.30 am** Breakfast	**8.30 am – 9.30 am** Breakfast	**8.30 am – 9.30 am** Breakfast	**8.30 am – 9.30 am** Breakfast	**8.30 am – 9.30 am** Breakfast
**9.30 am – 11.30 am** Exercise Program	**9.30 am – 11.30 am** Exercise Program	**9.30 am – 11.30 am** Exercise Program	**9.30 am – 11.30 am** Exercise Program	**9.30 am – 11.30 am** Rest day	**9.30 am – 11.30 am** Exercise Program	**9.30 am – 11.30 am** Exercise Program
**12.00 pm – 1.00 pm** Lunch	**12.00 pm – 1.00 pm** Lunch	**12.00 pm – 1.00 pm** Lunch	**12.00 pm – 1.00 pm** Lunch	**12.00 pm – 1.00 pm** Lunch	**12.00 pm – 1.00 pm** Lunch	**12.00 pm – 1.00 pm** Lunch
**1.00 pm – 2.00 pm** Leisure Time	**1.00 pm – 2.00 pm** Leisure Time	**1.00 pm – 6.00 pm** Medical health check	**1.00 pm – 2.00 pm** Leisure Time	**1.00 pm – 6.00 pm** Rest day	**1.00 pm – 2.00 pm** Leisure Time	**1.00 pm – 2.00 pm** Leisure Time
**2.00 pm – 3.00 pm** Presentation “Health behavior maintenance”	**2.00 pm – 4.00 pm** Exercise Program		**2.00 pm – 6.00 pm** Hiking Trip		**2.00 pm – 6.00 pm** Implementation strategies for home	**2.00 pm – 4.00 pm** Exercise Program
**3.00 pm – 6.00 pm** Leisure Time	**4.00 pm – 5.00 pm** Presentation “Barrier management”					**4.00 pm – 6.00 pm** Official farewell
	**5.00 pm – 6.00 pm** Leisure Time					
**6.00 pm – 7.00 pm** Dinner	**6.00 pm – 7.00 pm** Dinner	**6.00 pm – 7.00 pm** Dinner	**6.00 pm – 7.00 pm** Dinner	**6.00 pm – 7.00 pm** Dinner	**6.00 pm – 7.00 pm** Dinner	**6.00 pm – 7.00 pm** Dinner
**From 7.00 pm** Individual Coaching	**From 7.00 pm** Individual Coaching	**From 7.00 pm** Individual Coaching	**From 7.00 pm** Individual Coaching	**From 7.00 pm** Leisure Time	**From 7.00 pm** Individual Coaching	**From 7.00 pm** Departure
